# Ovitrap surveillance of dengue vector mosquitoes in Bandung City, West Java Province, Indonesia

**DOI:** 10.1371/journal.pntd.0009896

**Published:** 2021-10-28

**Authors:** Hadian Iman Sasmita, Kok-Boon Neoh, Sri Yusmalinar, Tjandra Anggraeni, Niann-Tai Chang, Lee-Jin Bong, Ramadhani Eka Putra, Amelia Sebayang, Christina Natalina Silalahi, Intan Ahmad, Wu-Chun Tu

**Affiliations:** 1 Department of Entomology, National Chung Hsing University, Taichung, Taiwan; 2 Center for Isotopes and Radiation Application, National Nuclear Energy Agency, Jakarta, Indonesia; 3 School of Life Sciences and Technology, Institut Teknologi Bandung, Bandung, West Java, Indonesia; 4 Department of Plant Medicine, National Pingtung University of Science and Technology, Pingtung, Taiwan; Federal University of Ceará, Fortaleza, Brazil, BRAZIL

## Abstract

Larval surveillance is the central approach for monitoring dengue vector populations in Indonesia. However, traditional larval indices are ineffective for measuring mosquito population dynamics and predicting the dengue transmission risk. We conducted a 14-month ovitrap surveillance. Eggs and immature mosquitoes were collected on a weekly basis from an urban village of Bandung, namely Sekejati. Ovitrap-related indices, namely positive house index (PHI), ovitrap index (OI), and ovitrap density index (ODI), were generated and correlated with environmental variables, housing type (terraced or high-density housing), ovitrap placement location (indoor or outdoor; household or public place), and local dengue cases. Our results demonstrated that *Aedes aegypti* was significantly predominant compared with *Aedes albopictus* at each housing type and ovitrap placement location. Ovitrap placement locations and rainfall were the major factors contributing to variations in PHI, OI, and ODI, whereas the influences of housing type and temperature were subtle. Indoor site values were significantly positively correlated to outdoor sites’ values for both OI and ODI. OI and ODI values from households were best predicted with those from public places at 1- and 0-week lags, respectively. Weekly rainfall values at 4- and 3-week lags were the best predictors of OI and ODI for households and public places, respectively. Monthly mean PHI, OI, and ODI were significantly associated with local dengue cases. In conclusion, ovitrap may be an effective tool for monitoring the population dynamics of *Aedes* mosquitoes, predicting dengue outbreaks, and serving as an early indicator to initiate environmental clean-up. Ovitrap surveillance is easy for surveyors if they are tasked with a certain number of ovitraps at a designated area, unlike the existing larval surveillance methodology, which entails identifying potential breeding sites largely at the surveyors’ discretion. Ovitrap surveillance may reduce the influence of individual effort in larval surveillance that likely causes inconsistency in results.

## Author summary

The dengue virus, transmitted by *Aedes* vectors, has been continuously spreading in tropical and subtropical countries, causing illness and fatality. Given the lack of a cost-effective dengue vaccine, the vector control approach for reducing the *Aedes* population remains the key method for mitigating dengue transmission. For a successful vector control program, an effective vector surveillance system is crucial for precisely predicting the spatial and temporal risk of a dengue outbreak. The ovitrap system improves data collection efficiency, aiding long-term dengue vector monitoring activities. This study is one of the few long-term dengue vector surveillance programs in Indonesia and provides compelling evidence of the need to improve the existing conventional larval surveillance system. The results demonstrated that two dengue vector mosquitoes, *A*. *aegypti* and *A*. *albopictus*, were present in the study area, and *A*. *aegypti* was more prevalent than *A*. *albopictus*. We observed an interactive relationship between ovitrap placement and rainfall in the dynamics of ovitrap-related indices; understanding this relationship allows for timely initiation of vector control and intervention strategies. We conclude that the ovitrap surveillance system is a sensitive tool for monitoring the population dynamics of *Aedes* vectors, predicting dengue outbreaks, and potentially improving community-based conventional larval surveillance.

## Introduction

Dengue infection is endemic in 90% of the districts and cities in Indonesia [1]. Although various vector control programs have been implemented since 1968, primarily by the Ministry of Health, the data demonstrate that the nationwide spread of dengue infection sharply increased the annual incidence of dengue hemorrhagic fever (DHF) over 50 years from just 0.05 cases per 100,000 persons per year in 1968 to 77.96 cases per 100,000 persons per year in 2016 [2]. Unlike in previous years, where dengue cases usually subsided toward the end of the wet season in March, the dengue case outbreak in 2020 lasted until June, registering 100–500 cases per day [3]. However, this change in the epidemic pattern does not portend a future trend, but it does warn that dengue prevalence may get worse if not controlled.

Vector surveillance is crucial for determining the distribution, population density, larval habitats, and spatiotemporal risk factors related to dengue transmission [4]. In most countries, vector surveillance is largely based on larval surveys [5]. In Indonesia, the house index (the percentage of houses infested with larvae or pupae), container index (the percentage of water-holding containers infested with larvae or pupae), Breteau index (the number of positive containers per 100 houses inspected) [6–8], and new larvae-free index (local name: Angka Bebas Jentik; the percentage of premises not infested with larvae or pupae) have been used as indicators of dengue transmission risk [9]. However, the aforementioned Stegomyia indices have been inadequate for estimating dengue vector abundance [5] and dengue transmission risk [10]. For instance, Wijayanti et al. [11], who conducted a dengue vector surveillance study in Banyumas regency, West Java, Indonesia, provided compelling evidence that traditional larval indices are inadequate indicators for predicting dengue transmission risk, partly because of disparities in the vectorial capacity of *Aedes* mosquitoes across regions and shortcomings of the diagnostic tools. In Indonesia, the vast area and regional disparities in terms of health and socioeconomic status further complicate the problem. Vector surveillance has usually been implemented for a short period, and sampling techniques have not been standardized among surveyed areas owing to a shortage of funds.

The ovitrap surveillance system is an alternative for long-term vector surveillance to provide insight regarding population dynamics and the spatiotemporal distribution of mosquito vectors for improving dengue prevention and control programs. Ovitrap, an inexpensive, easy-to-use, and effective tool for monitoring dengue vectors [5], has been used for routine surveillance for dengue in Hong Kong [12], Singapore [13], Taiwan [14], and Australia [15]. Ovitrap surveying is preferable to larval surveys because it is an active surveillance method that detects not only immature mosquitoes but also eggs laid by gravid mosquitoes [16,17]. Manica et al. [18] estimated that for every five eggs in an ovitrap, one person gets bitten by a female *Aedes*. In southern Taiwan, >3000 ovitraps were set annually at public and residential areas in Tainan City and Kaohsiung City. Entomological data derived from ovitrap surveillance provide a benchmark for mobilizing environmental clean-ups and reducing mosquito breeding sites; if an ovitrap contains >500 eggs or the ovitrap index (OI) is >60%, environmental clean-up is essential. The surveillance system has been effective in curbing outbreaks in Taiwan [19,20]. However, using ovitrap data has several limitations. Wijegunawardana et al. [17] argued that the estimation obtained through ovitrap surveys may not accurately reflect gravid mosquito abundance when natural or artificial breeding sites in a given area are abundant. Not all gravid mosquitoes may oviposit their eggs in an oviposition site but may deposit them in other water-holding containers. This may result in female abundance underestimation. The number of eggs deposited in ovitraps does not necessarily represent the abundance of biting female mosquitoes [21]. OI alone may not be suitable for predicting dengue outbreaks, and environment, mosquito biology, and the socioeconomic status of local residents should be considered [19,22,23].

In this study, we conducted ovitrap surveillance on a weekly basis for 14 months. A series of indices based on this surveillance, namely the positive house index (PHI; the proportion of houses with positive ovitraps), OI (the proportion of ovitraps containing *Aedes* egg or immature mosquitoes), and ovitrap density index (ODI; the average number of *Aedes* eggs per positive ovitrap), were generated to correlate with environmental variables, housing type, ovitrap placement location, and local dengue cases. In particular, our goal was to address whether (1) vector indices differ between ovitrap placement locations (i.e., indoor vs. outdoor and terraced vs. high-density housing) in dry and wet seasons, (2) vector indices differ between households and public places in dry and wet seasons, (3) vector indices covary with the number of dengue cases at the study site, and (4) environmental data at the weekly lag period could be used to predict vector indices of households and public areas. On the basis of outcomes, we aimed to formulate an effective vector surveillance system to improve the data collection efficiency.

## Methods

### Ethics statement

The surveillance protocol was approved by the Bandung City government through the Agency of National Unity and Politics (Badan Kesatuan Bangsa dan Politik) as registered in NOMOR: 070/2196/XII-2019/BKBP. Verbal informed consent was obtained from the household heads of all households agreeing to participate in entomological and ovitrap surveys.

### Study area

**Fig 1:**
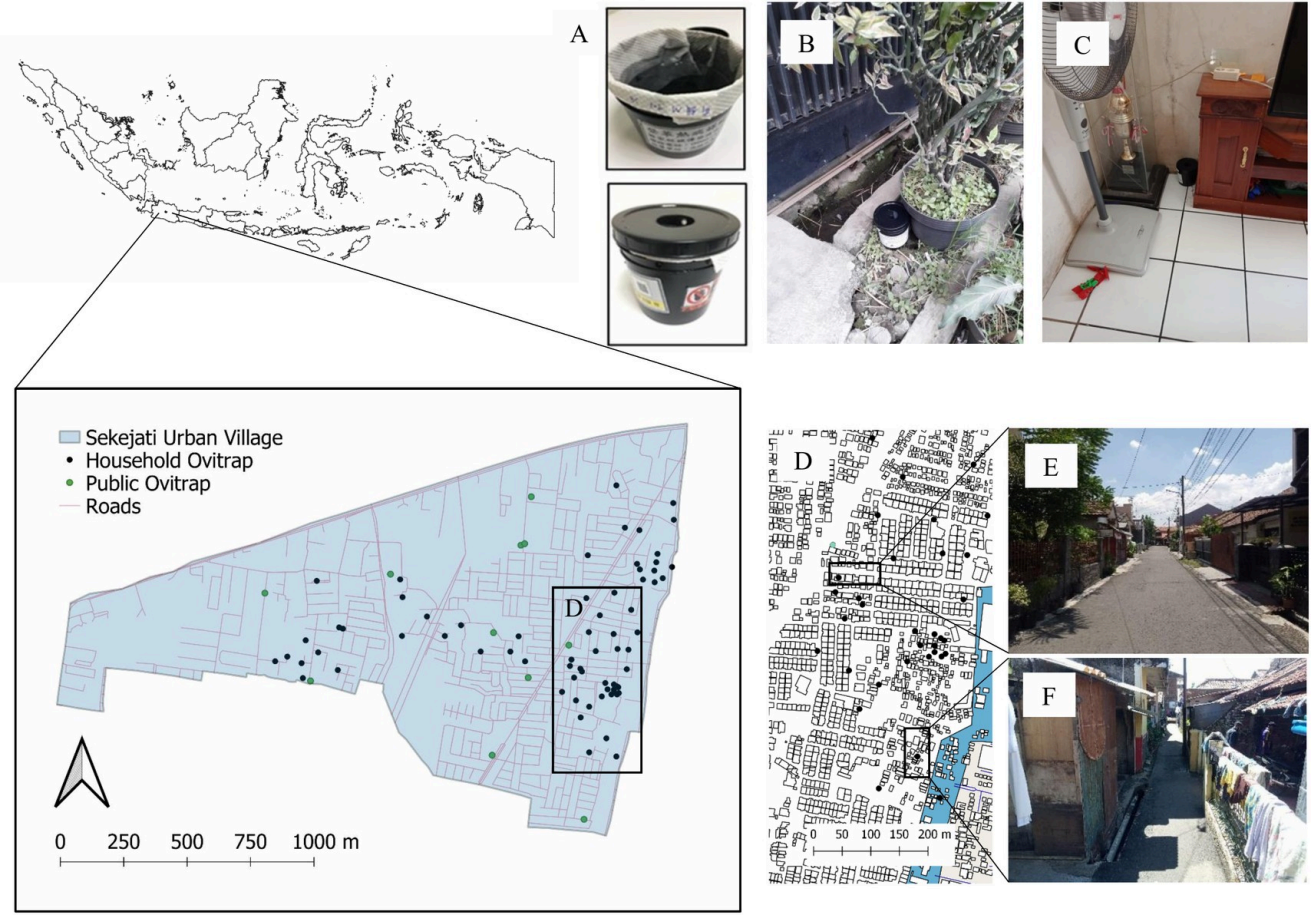
Ovitrap distribution in Sekejati urban village, Bandung. Solid black circles indicate ovitraps installed at households, whereas solid green circles indicate ovitraps installed at public places. (A) Ovitrap with its oviposition substratum. Ovitrap placement at a (B) public place and (C) household. (D) Household arrangement, with a road separating blocks in (E) terraced housing and (F) high-density housing.

Sekejati urban village, with a total area of 195.14 ha (6°56′48.38″S, 107°39′41.92″E), located in Buahbatu subdistrict, Bandung, West Java, Indonesia, was selected for the study (Fig 1). Sekejati is one of the high-risk areas of dengue transmission in Bandung City, with 193 DHF cases registered from January 2017 to March 2020 (source: Dinas Kesehatan Kota Bandung 2020). The human settlement of Sekejati encompasses 146.35 ha (75% of the total area) with 6,425 households (source: Sekejati administration office 2020). The study area has two types of dwellings: (1) terraced housing, where rows of houses are separated with 6–7-m-wide asphalt roads, and (2) high-density housing, where houses are situated close to each other, separated by 1–3-m-wide asphalt roads (Fig 1D, 1E, and 1F).

### Ovitrap surveillance

Ovitrap surveys were conducted at terraced and high-density housing areas on a weekly basis from September 2018 to October 2019 (60 weeks). An ovitrap consists of a black plastic container (diameter: 10.6 cm; height: 13.5 cm) with an aperture (diameter: 4 cm) at the center of the lid. The inner walls of ovitraps were lined with two kitchen towels (24 cm × 11.5 cm; Tessa Soft Hand Towel THSN-001, Bekasi, Indonesia), with two-thirds of the ovitrap filled with water as a female *Aedes* oviposition site (Fig 1A). For household placement of ovitraps, an outdoor ovitrap was installed in a shady corner of a veranda, and two indoor ovitraps were installed in a bathroom/kitchen (wet area) and living room/bedroom (dry area). Thus, 96 and 114 ovitraps were installed at 32 terraced houses and 38 high-density houses, respectively. Indices derived from 10 public places, including schools, mosques, and public parks, were compared with indices derived from households. Three ovitraps were installed at each public place. Each ovitrap was assigned a serial number, house address, and Global Positioning System coordinates (Garmin eTrex 30×; Garmin, Olathe, KS, USA) to facilitate data collection and management (Fig 1B and 1C).

### Mosquito collection and rearing

Mosquito eggs, larvae, and pupae in ovitraps were collected weekly. However, if participating residents were unavailable, the collection was conducted after 8–9 days. In brief, oviposition strips (kitchen towels) were folded and kept in a ziplock bag. All larvae or pupae found in an ovitrap were counted, and all specimens were transported to the laboratory (Komplek PPR research station, Institute Teknologi Bandung, Bandung City, West Java Province) for rearing until their adult stage for species identification. All hatched larvae were reared using aged water with ad libitum fish food pellets (Takari, CPPETINDO, Mojokerto, Indonesia) until pupation at 25°C ± 1°C, relative humidity of 60% ± 5%, and photoperiod 12:12. Species were identified using a light microscope.

### Vector indices

Three vector indices were used in this study, namely PHI, OI, and ODI. The PHI of households was determined by dividing the number of houses with positive ovitraps by the total number of houses examined, whereas the PHI of public places was calculated by dividing the number of public places with positive ovitraps by the total number of public places examined. The OI was determined by dividing the number of ovitraps containing *Aedes* egg or immature mosquitoes by the total number of ovitraps observed. The ODI is the average number of *Aedes* eggs per positive ovitrap.

### Meteorological data

Daily temperature and rainfall data were obtained from the Bandung Geophysics Station of the Meteorological, Climatological, and Geophysical Agency (6°53′00.10″S, 107°35′49.91″E). The distance between the meteorological station and the study site was approximately 4.8 km. Rainfall data were collected weekly, coinciding with weekly ovitrap data collection, for further analysis.

### Data analysis

The percentage abundance of each species was calculated by dividing the total number of specimens by the total number of species. Vector indices were calculated based on the season and ovitrap placements for each week. All analyses were conducted using SPSS version 18.0 (SPSS Inc., Chicago, IL, USA) at α = 0.05.

#### Effect of season, ovitrap placement location, and housing type on vector indices

To examine the effect of season, indoor/outdoor ovitrap placement, housing type, household/public location, and their interaction on the presence of a positive ovitrap and positive house, a generalized linear model with logistic distribution was used. Furthermore, to examine the aforementioned effects on the number of eggs in a positive ovitrap, a generalized linear model with a negative binomial distribution was used to correct overdispersion. Indoor/outdoor ovitrap placement was not considered in the determination of positive houses owing to the lack of statistical reasoning. This is because a positive house was determined on the basis of a positive ovitrap being present in a house regardless of its placement indoors or outdoors. To address whether the OI and ODI of indoor ovitraps covary with those of outdoor ovitraps, the correlation between the two was investigated through regression analysis. In addition, a regression analysis was used to examine how the vector indices of household ovitraps are associated with those of public areas at varying lag periods.

#### Association of vector indices with number of dengue cases at study site

A regression analysis was used to correlate vector indices of households and public places with the number of dengue cases. Monthly dengue cases were obtained from the Bandung Health Office.

#### Association of cumulated rainfall and average temperature of 0–4-week lags with vector indices of household and public areas

A regression analysis was used to determine the predictive power of the weekly cumulative rainfall and average temperature of varying lag periods, which could account for a significant proportion of the variance in the regression model of vector indices from households and public areas.

## Results

**Fig 2:**
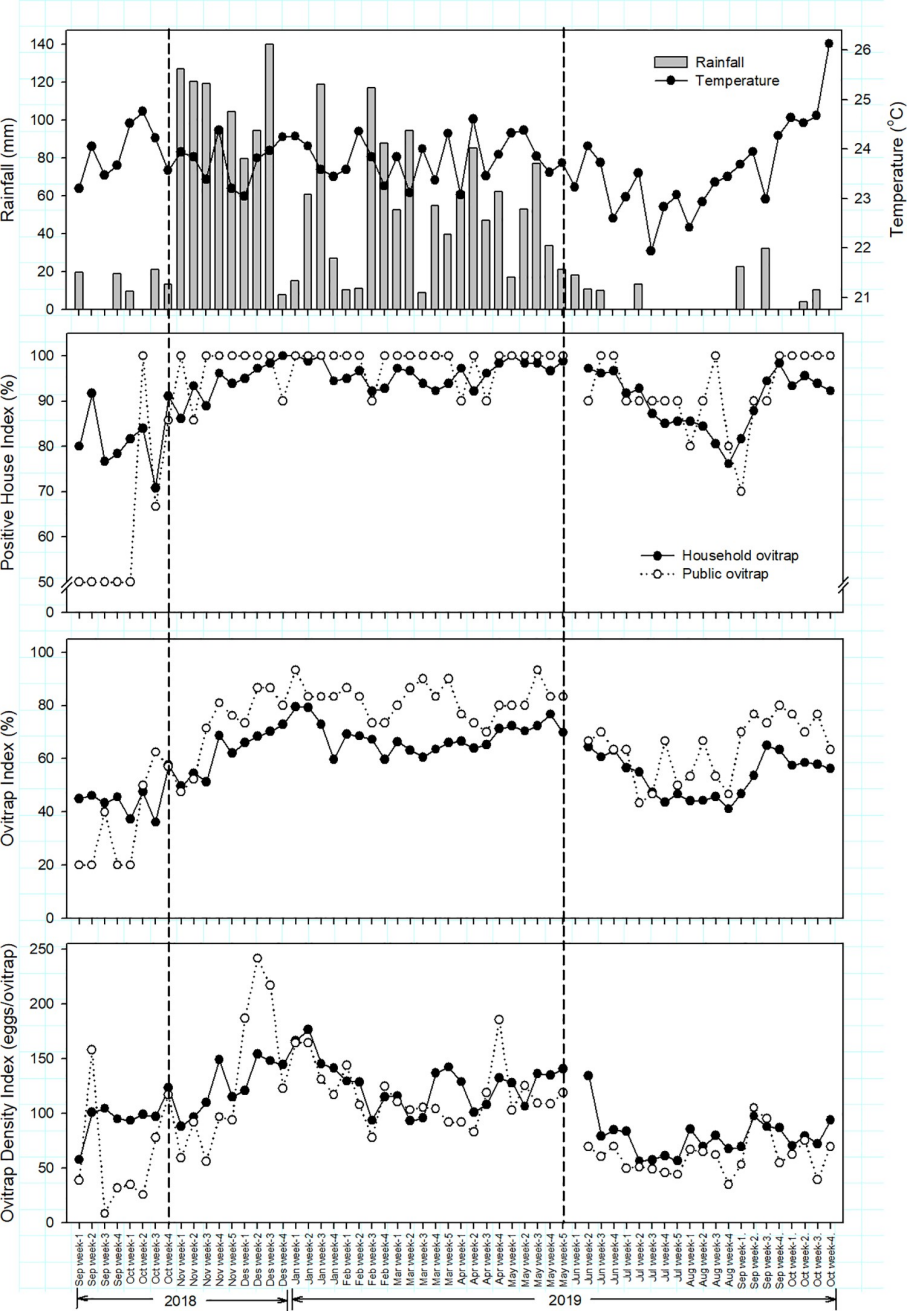
*Aedes* vector indices of ovitraps installed at households and public places in Sekejati urban village, Bandung, from September 2018 to October 2019. The cumulative rainfall of the wet season was calculated from the 4th week of October 2018 to the 5th week of May 2019.

During 2018–2019, Sekejati experienced an average temperature of 23.7°C (max: 24.8°C; min: 22.8°C) and monthly average precipitation of 167.76 mm, with August 2019 being the driest month (0.2 mm) and November 2018 the wettest month (483.2 mm). Two dry spells occurred during the study, which lasted from early September 2018 to mid-October 2018 and from early June 2019 to October 2019, whereas the rainy season spanned from the end of October 2018 to the end of May 2019 (Fig 2).

### Percentage of dengue vector abundance

A total of 806,102 mosquito eggs were captured throughout the sampling period, with an average of 175.24 (±12.68) and 207.4 (±17.24) eggs per ovitrap per week collected from households and public places, respectively. Two dengue vectors, namely *A*. *aegypti* and *A*. *albopictus*, were identified at the study site, with *A*. *aegypti* predominantly found in households and public places (Table 1). Specifically, the percentage abundance of *A*. *aegypti* relative to *A*. *albopictus* was 98.1% (n = 156,115) in households and 86.1% (n = 20,800) in public places. *A*. *aegypti* was abundant in both indoor (99.4%, n = 62,481) and outdoor (97.3%, n = 93,634) locations compared with *A*. *albopictus*. Similarly, in both housing types, *A*. *aegypti* eggs were more abundant than *A*. *albopictus* eggs, accounting for 97.8% (n = 73,899) in terraced housing and 98.7% (n = 76,335) in high-density housing.

**Table 1:** Number and percentage abundance of dengue vector mosquitoes that hatched from eggs collected from various ovitrap placements.

Ovitrap placement	Number of specimen (% abundance)	
	*A*. *aegypti*	*A*. *albopictus*
Households	156,115 (98.1)	2,992 (1.9)
Indoor	62,481 (99.4)	365 (0.6)
Outdoor	93,634 (97.3)	2,627 (2.7)
Public places	20,800 (86.1)	3,340 (13.9)
Terraced housing	73,899 (97.8)	1,645 (2.22)
High density housing	76,335 (98.7)	1,002 (1.3)

### Vector indices between ovitrap placement locations in dry and wet seasons

Based on the odds ratio from binary logistic regression in terms of PHI in the terraced/high-density housing category (Table 2), the odds of a house having a positive ovitrap in the wet season were 3.4 times those of a house having a positive ovitrap in the dry season (P < 0.0001) when controlling for all variables. Other terms from this category were not significant. Similarly, the odds of a house/public place having a positive ovitrap in the wet season were 3 times higher than those in the dry season. Additionally, the odds of a public place having a positive ovitrap were 6.4 times higher than those of a house (P < 0.0001). The interaction between the dry/wet season and household/public place placement was nonsignificant.

**Table 2:** Results of generalized linear model analysis of effects of seasonality, ovitrap placement location, and housing type on vector indices. PHI and OI were subjected to binary logistic regression, whereas ODI was subjected to negative binomial regression. Explanatory variables in bold were considered as the reference.

Response variables	Explanatory variables	Coefficient (B)	S.E.	95% C.I.		Statistical value	P-value	Exp(B)
				Lower	Upper			
	*Dry/wet season vs Indoor/outdoor placement vs Terraced/high density housing*							
PHI	Intercept	-3.084	0.142	-3.362	-2.806	472.934	<0.0001	0.046
	**Dry**/wet season	1.232	0.170	0.898	1.565	52.425	<0.0001	3.428
	**Terraced**/high density	-0.043	0.212	-0.459	0.372	0.041	0.839	0.958
	**Dry**/wet season * **terraced**/high density	-0.157	0.258	-0.660	0.346	0.373	0.541	0.855
OI	Intercept	-1.627	0.078	-1.780	-1.473	431.244	<0.0001	0.197
	**Dry**/wet season	0.631	0.107	0.421	0.840	34.876	<0.0001	1.879
	**Indoor**/outdoor	1.432	0.088	1.259	1.606	261.781	<0.0001	4.187
	**Terraced**/high density	-0.053	0.117	-0.282	0.177	0.204	0.652	0.948
	**Dry**/wet season * **Indoor**/outdoor	0.017	0.124	-0.225	0.260	0.019	0.889	1.017
	**Dry**/wet season * **Terraced**/high density	0.144	0.158	-0.166	0.453	0.829	0.363	1.155
	**Indoor**/outdoor * **Terraced**/high density	-0.076	0.132	-0.335	0.183	0.330	0.565	0.927
	**Dry**/wet season * **Indoor**/outdoor * **Terraced**/high density	-0.167	0.183	-0.525	0.191	0.837	0.360	0.846
ODI	Intercept	-4.910	0.032	-4.973	-4.848	23719.669	<0.0001	0.007
	**Dry**/wet season	0.468	0.050	0.371	0.565	89.284	<0.0001	1.597
	**Indoor**/outdoor	0.373	0.042	0.290	0.456	77.772	<0.0001	1.452
	**Terraced**/high density	0.186	0.047	0.094	0.278	15.646	<0.0001	1.204
	**Dry**/wet season * **Indoor**/outdoor	-0.088	0.068	-0.221	0.045	1.692	0.193	0.916
	**Dry**/wet season * **Terraced**/high density	0.012	0.073	-0.131	0.156	0.029	0.865	1.012
	**Indoor**/outdoor * **Terraced**/high density	-0.390	0.062	-0.512	-0.268	39.395	<0.0001	0.677
	**Dry**/wet season * **Indoor**/outdoor * **Terraced**/high density	-0.026	0.099	-0.220	0.169	0.067	0.795	0.974
	*Household vs Public*							
PHI	Intercept	-1.248	0.144	-1.531	-0.966	75.046	<0.0001	0.287
	**Dry**/wet season	1.101	0.198	0.713	1.489	30.923	<0.0001	3.007
	**Household**/public place	-1.855	0.178	-2.205	-1.505	108.001	<0.0001	0.156
	**Dry**/wet season * **household**/public place	0.062	0.235	-0.399	0.524	0.070	0.791	1.064
OI	Intercept	-1.358	0.088	-1.530	-1.187	240.764	<0.0001	0.257
	**Dry**/wet season	0.771	0.121	0.533	1.009	40.307	<0.0001	2.162
	**Household**/public place	0.718	0.091	0.539	0.897	61.766	<0.0001	2.050
	**Dry**/wet season * **household**/public place	-0.174	0.127	-0.423	0.075	1.878	0.171	0.840
ODI	Intercept	-4.795	0.040	-4.873	-4.717	14548.741	<0.0001	0.008
	**Dry**/wet season	0.629	0.064	0.503	0.755	95.522	<0.0001	1.876
	**Household**/public place	0.071	0.043	-0.013	0.154	2.766	0.096	1.074
	**Dry**/wet season * **household**/public place	-0.216	0.069	-0.351	-0.081	9.827	0.002	0.806

When considering positive ovitraps in indoor/outdoor and terraced/high-density housing locations (Table 2), the odds of an ovitrap being infested with *Aedes* eggs in the wet season were 1.9 times higher than those in the dry season (P < 0.0001) when controlling for all variables. Furthermore, the indoor/outdoor placement in a house was a significant factor associated with the positivity rate of an ovitrap. For instance, the probability of an outdoor ovitrap having eggs was 4.2 times higher than that of an indoor ovitrap (P < 0.0001). Other terms from this category, including interactions, were not significant. When considering positive ovitraps in household/public locations, the odds of an ovitrap having eggs were 2.2 times higher in the wet season than in the dry season (P < 0.0001). Ovitraps in public places were twice as likely to be infested by *Aedes* compared with those in households (P < 0.0001). The effect of the interaction between seasonality and household/public location on a positive ovitrap was nonsignificant.

Negative binomial regression revealed that the nominal effects of the dry/wet season, indoor/outdoor placement, and housing type were significantly associated with the number of eggs in ovitraps independent of other parameters (P < 0.0001), but the household/public placement was not associated with the number of eggs in ovitraps (P = 0.096; Table 2). The number of eggs per ovitrap in the wet season was 59.7% higher than in the dry season (P < 0.0001). Outdoor ovitraps contained approximately 45.2% more eggs than did indoor ovitraps (P < 0.0001). The number of eggs in terraced housing was 20.4% higher than in high-density housing (P < 0.0001). A statistically significant interaction indicated that the effects of housing type on ODI depend on ovitrap placement in terms of the indoor/outdoor variable.

When considering the associations between vector indices, significant positive correlations of OI (r_(58)_ = 0.6938, P < 0.0001) and ODI (r_(58)_ = 0.7066, P < 0.0001) were observed between indoor and outdoor ovitraps (S1 Fig). A linear regression analysis demonstrated that the increasing trend of OI of households was best predicted by the OI of public places in the same week and in the 1-week lag period (66.0% of variance explained). The ODI of public places explained 52% of the total variance of ODI of households in the same week (Table 3).

**Table 3:** Regression analysis of vector indices of weekly household and public places at 0–4-week lag periods.

Response variable	Predictor variable	r^2^	df	n	F	*P*-value
OI household	OI public lag 0 week	0.6558	1	59	110.5026	<0.0001
	OI public lag 1 week	0.6593	1	58	110.3085	<0.0001
	OI public lag 2 weeks	0.5667	1	57	73.2328	<0.0001
	OI public lag 3 weeks	0.4678	1	56	48.3394	<0.0001
	OI public lag 4 weeks	0.3764	1	55	32.5969	<0.0001
ODI household	ODI public lag 0 week	0.5164	1	59	61.9434	<0.0001
	ODI public lag 1 week	0.4103	1	58	39.6660	<0.0001
	ODI public lag 2 weeks	0.3064	1	57	24.7338	<0.0001
	ODI public lag 3 weeks	0.3606	1	56	31.0204	<0.0001
	ODI public lag 4 weeks	0.3240	1	55	25.8824	<0.0001

### Association between vector indices and number of dengue cases at the study site

**Fig 3:**
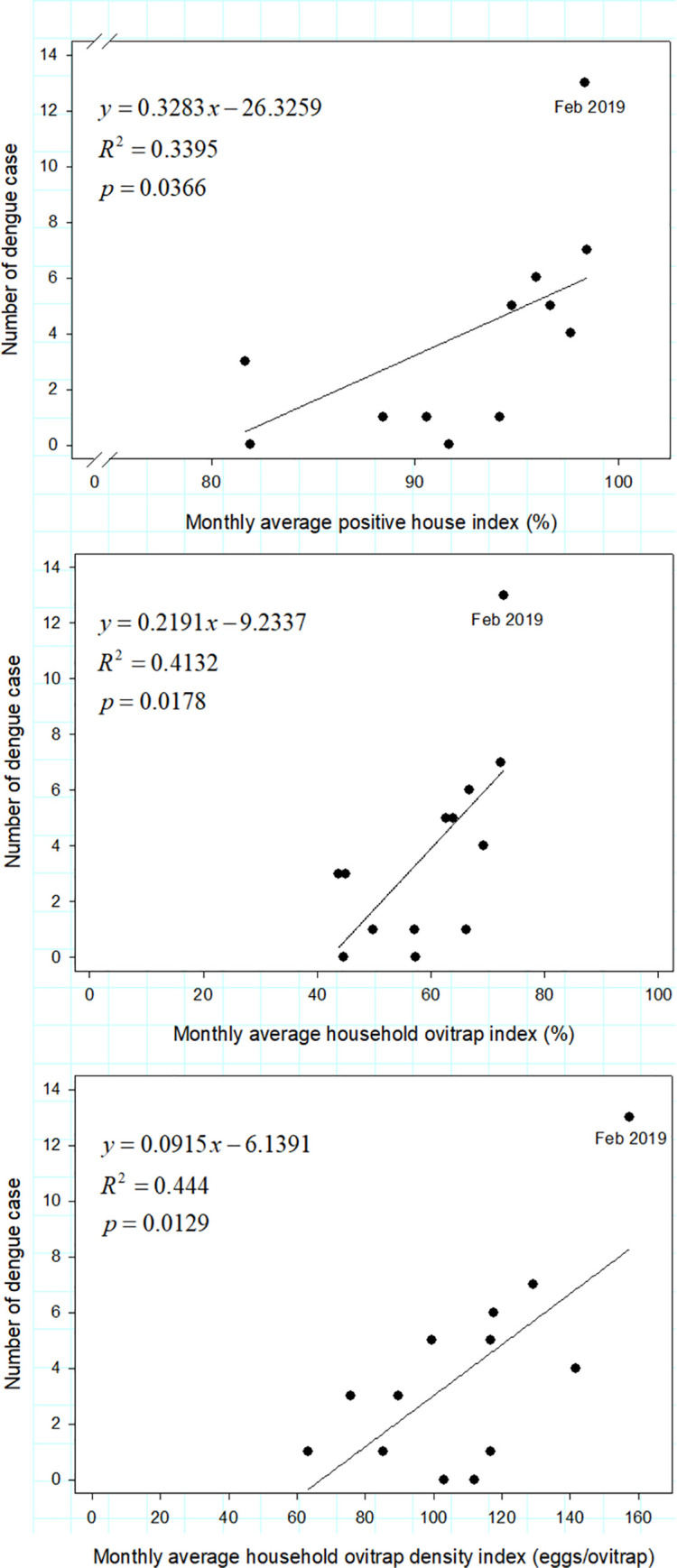
Regression analysis of monthly PHI, OI, and ODI of households and monthly dengue cases reported in Sekejati urban village, Bandung City.

Monthly dengue cases in Sekejati urban village peaked in February 2019 (Fig 3). The monthly dengue cases were positively associated with the monthly average PHI (r_(13)_ = 0.5827, P = 0.0366), OI (r_(13)_ = 0.6428, P = 0.0178), and ODI (r_(13)_ = 0.6664, P = 0.0129) of households. The number of dengue cases was best explained by the monthly mean ODI and OI, which explained 44.4% and 42.32% of the variation, respectively; whereas PHI explained 33.95% of the variation (Fig 3). By contrast, the monthly dengue cases were not significantly associated with monthly average indices of public places [PHI (r_(13)_ = 0.3369, P = 0.2604), OI (r_(13)_ = 0.4756, P = 0.1004), and ODI (r_(13)_ = 0.5194, P = 0.0689)].

### Weekly total rainfall and average temperature to predict vector indices in households and public areas

Among significant predictors, the weekly total rainfall in the 3-week lag period best predicted the OI and ODI of households, explaining 40% and 43% of the variation compared with the weekly total rainfall in the same week and 1-, 2-, and 4-week lag periods. Similarly, the weekly rainfall in the 3-week lag period best explained the OI and ODI of public places, explaining 31% and 39% of the variation, respectively (Table 4).

**Table 4:** Regression analysis of weekly total rainfall and mean temperature at 0–4-week lag periods in relation to vector indices of households and public areas.

Response variable	Predictor variable	r^2^	df	n	F	*P*-value
PHI household	Weekly total rainfall lag 0	0.0977	1	58	6.1720	0.0159
	Weekly total rainfall lag 1	0.1918	1	57	13.2895	0.0006
	Weekly total rainfall lag 2	0.2177	1	56	15.3064	0.0003
	Weekly total rainfall lag 3	0.2804	1	55	21.0408	<0.0001
	Weekly total rainfall lag 4	0.2734	1	54	19.9465	<0.0001
OI household	Weekly total rainfall lag 0	0.1424	1	58	9.4650	0.0032
	Weekly total rainfall lag 1	0.1693	1	57	11.4124	0.0013
	Weekly total rainfall lag 2	0.2841	1	56	21.8268	<0.0001
	Weekly total rainfall lag 3	0.4001	1	55	36.0202	<0.0001
	Weekly total rainfall lag 4	0.3732	1	54	31.5516	<0.0001
ODI household	Weekly total rainfall lag 0	0.1674	1	58	11.4610	0.0013
	Weekly total rainfall lag 1	0.2488	1	57	18.5491	<0.0001
	Weekly total rainfall lag 2	0.3432	1	56	28.7386	<0.0001
	Weekly total rainfall lag 3	0.4302	1	55	40.7661	<0.0001
	Weekly total rainfall lag 4	0.3078	1	54	23.5694	<0.0001
PHI public	Weekly total rainfall lag 0	0.0887	1	58	5.5500	0.0219
	Weekly total rainfall lag 1	0.0978	1	57	6.0702	0.0168
	Weekly total rainfall lag 2	0.1454	1	56	9.3583	0.0034
	Weekly total rainfall lag 3	0.1285	1	55	7.9648	0.0067
	Weekly total rainfall lag 4	0.0791	1	54	4.5534	0.0375
OI public	Weekly total rainfall lag 0	0.0983	1	58	6.2160	0.0156
	Weekly total rainfall lag 1	0.1532	1	57	10.1280	0.0024
	Weekly total rainfall lag 2	0.2870	1	56	22.1371	<0.0001
	Weekly total rainfall lag 3	0.3058	1	55	23.7899	<0.0001
	Weekly total rainfall lag 4	0.2314	1	54	15.9535	0.0002
ODI public	Weekly total rainfall lag 0	0.1925	1	58	13.5865	0.0005
	Weekly total rainfall lag 1	0.2198	1	57	15.7780	0.0002
	Weekly total rainfall lag 2	0.3367	1	56	27.9148	<0.0001
	Weekly total rainfall lag 3	0.3879	1	55	34.2207	<0.0001
	Weekly total rainfall lag 4	0.3276	1	54	25.8244	<0.0001
PHI household	Weekly mean temperature lag 0	0.0316	1	58	1.8587	0.1781
	Weekly mean temperature lag 1	0.0320	1	57	1.8509	0.1791
	Weekly mean temperature lag 2	0.0705	1	56	4.1723	0.0459
	Weekly mean temperature lag 3	0.1626	1	55	10.4891	0.0021
	Weekly mean temperature lag 4	0.1433	1	54	8.8640	0.0044
OI household	Weekly mean temperature lag 0	0.0394	1	58	2.3402	0.1316
	Weekly mean temperature lag 1	0.0715	1	57	4.3135	0.0424
	Weekly mean temperature lag 2	0.1119	1	56	6.9265	0.0110
	Weekly mean temperature lag 3	0.1430	1	55	9.0069	0.0041
	Weekly mean temperature lag 4	0.0996	1	54	5.8646	0.0189
ODI household	Weekly mean temperature lag 0	0.0581	1	58	3.5144	0.0660
	Weekly mean temperature lag 1	0.0741	1	57	4.4813	0.0387
	Weekly mean temperature lag 2	0.1340	1	56	8.5078	0.0051
	Weekly mean temperature lag 3	0.1367	1	55	8.5501	0.0050
	Weekly mean temperature lag 4	0.0754	1	54	4.3219	0.0425
PHI public	Weekly mean temperature lag 0	0.0137	1	58	0.7890	0.3781
	Weekly mean temperature lag 1	0.0055	1	57	0.3089	0.5806
	Weekly mean temperature lag 2	0.0306	1	56	1.7387	0.1928
	Weekly mean temperature lag 3	0.1928	1	55	3.5876	0.0636
	Weekly mean temperature lag 4	0.1278	1	54	7.7691	0.0074
OI public	Weekly mean temperature lag 0	0.0229	1	58	1.3363	0.2525
	Weekly mean temperature lag 1	0.0276	1	57	1.5910	0.2124
	Weekly mean temperature lag 2	0.0720	1	56	4.2671	0.0436
	Weekly mean temperature lag 3	0.0382	1	55	2.1440	0.1489
	Weekly mean temperature lag 4	0.0100	1	54	0.5340	0.4682
ODI public	Weekly mean temperature lag 0	0.0052	1	58	0.2988	0.5868
	Weekly mean temperature lag 1	0.0038	1	57	0.2139	0.6455
	Weekly mean temperature lag 2	0.0510	1	56	2.9535	0.0913
	Weekly mean temperature lag 3	0.0424	1	55	2.3911	0.1279
	Weekly mean temperature lag 4	0.0573	1	54	3.2230	0.0783

Although a significant association was identified between the weekly average temperature of the 1–4-week lag periods and vector indices of households, the trend of vector indices was not satisfactorily explained (PHI: 7%–16% of variation; OI: 7%–14% of variation; and ODI: 7%–14% of variation). In addition, only two of the weekly average temperatures of the 0–4-week lag periods were significantly correlated with the vector indices of public places and were not satisfactorily explained (PHI of lag 4: 13% of variation and OI of lag 2: 7% of variation; Table 4).

## Discussion

Long-term vector surveillance is crucial for developing prevention and mitigation strategies against dengue transmission. The present study provides information on the spatiotemporal distribution and population density of *Aedes* vectors obtained through ovitrap-related indices. These data may help health authorities, such as the Ministry of Health and local health offices, to improve current dengue entomological surveillance protocols that have been constrained by low-level community participation [24].

Sekejati urban village is a typical residential dwelling of a suburban area in Indonesia. The proportion of *A*. *aegypti* at public places was significantly low compared with that at households. It was reasoned that 6 of 10 public places chosen were adjacent to gardens with abundant vegetation—*A*. *aegypti* is significantly negatively associated with vegetation [25]. Although *A*. *albopictus* is generally found in areas with high vegetation cover, *A*. *albopictus* has been reported in highly dense urban or suburban areas in Italy [26], Taiwan [27], and Malaysia [28–30]. In Penang Island, Malaysia, *A*. *albopictus* was dominant in three areas of human dwelling [23]. Similarly, in Indonesia, the mixed infestation of *A*. *aegypti* and *A*. *albopictus* was reported in suburban regions of the provinces of Central Java [11], East Kalimantan, Bali, West Nusa Tenggara, and East Nusa Tenggara [31]. In our study, *A*. *albopictus*, in some conditions, shared the same ovitraps with *A*. *aegypti* as oviposition sites at household indoor and outdoor locations, although the probability was low. This finding indicates that *A*. *albopictus* has high plasticity in choosing oviposition sites for laying eggs in natural as well as artificial containers in urban green areas and highly urbanized habitat with less vegetation [32]. Furthermore, *A*. *albopictus* is an opportunistic biter and prefer to feed on human blood rather than animal blood [33], although its feeding behavior varies according to the geographic origin of the mosquito population [32].

In the present study, *A*. *aegypti* was found predominantly in households, and outdoor ovitraps were more likely to contain mosquito eggs than were indoor ovitraps. The water-holding containers scattered outside houses attract female *A*. *aegypti* to oviposit. Wijayanti et al. [11], who used conventional larval indice surveillance at four villages in Central Java, documented that the proportions of outdoor infestation in discarded tires (53%) and flower pots (26%) were high. Furthermore, the results somewhat agree with the finding of Martin et al. [34], who surveyed *A*. *aegypti* at indoor and outdoor household locations by using autocidal ovitraps in South Texas and noted that outdoor ovitraps had a higher relative abundance than did indoor ovitraps. However, in the dry season, the mean OI and ODI of indoor locations remained high, comprising 40% and 72 eggs, respectively (S1 Table). One of the potential explanations for the result is that in local residential dwelling areas, it is a custom to store water in buckets for daily consumption and cooking and in water tanks for bathing [11]. In the wet season, the OI and ODI of indoor locations increased in parallel with those of outdoor locations (S1 Fig). The significant correlations of OI and ODI between indoor and outdoor ovitraps may be indicative of the high mobility of *Aedes* adults flying in and out of the house. By contrast, Martin et al. [34] documented that no significant seasonal changes occurred in an indoor mosquito population.

The current study examined whether the socioeconomic status of the community at study sites, to a certain extent, affects dengue vector abundance. In general, high-density housing may reflect low household income and educational levels, whereas terraced housing may reflect the opposite (S2 Table). We posit that the mosquito population in high-density housing is higher than that in terraced housing due to poor housing conditions and drainage systems and the presence of cemeteries, communal sanitation facilities, improper garbage management, and abandoned lands, which primarily contribute to increased dengue vector indices in high-density housing relative to terraced housing [22]. The hypothesis was supported by our result that the odds of ODI in high-density housing were significantly higher than in terraced housing (Table 2). The finding is further supported by a study in Texas, USA, which revealed that the indoor mosquito population was significantly greater in low-income communities than in middle-income communities [34]. However, no significant difference was observed in PHI and OI between terraced housing and high-density housing in the current study. The result suggested that instead of poor sanitation in high-density housing, potted plants, fish ponds, and storage of unused possessions on verandas in terraced housing may have equally contributed to *Aedes* mosquito breeding.

The effectiveness of the ovitrap surveillance system in improving dengue vector management has been evaluated in Taiwan [14,19,27], Malaysia [23,29,30,35–37], Trinidad [38], and the Philippines [39]. Our results indicated that the monthly average PHI, OI, and ODI values were significantly correlated with the monthly total local dengue incidence. However, several caveats to the present study are worth considering. For instance, the reported dengue cases were based on the patient’s residence, but an individual’s house might not necessarily be the only place where they can contract the disease; other possibilities include working places and recreational areas. A nondefinitive dengue fever diagnostic protocol is one potential problem contributing to type II error. In Indonesia, immunochromatographic rapid tests and reverse transcription polymerase chain reaction, which are sensitive tools for confirming dengue infection, are rarely available at primary health facilities, and hence, diagnoses are typically based on clinical presentation and a common laboratory evaluation, which leaves many dengue cases undetected [24,40].

As a rule, rainfall is a major variable for forecasting increases in the dengue vector population. Rain fills natural and artificial water-holding containers, creating suitable habitats for oviposition and larval development, which leads to a high adult population in the environment as well as in residential dwellings. In Brazil, precipitation was directly related to an increase in *Aedes* spp. eggs in ovitraps [41]. A study in Italy on *A*. *albopictus* showed that female abundance was significantly associated with 1–4 weeks of accumulated rainfall [42]. Weekly BG-Sentinel counts capturing adult *Aedes* spp. were positively associated with rainfall in the preceding month in Yogyakarta, Indonesia [25]. A direct association between rainfall and dengue incidence has been reported in several Asian countries [11,43,44]. Our study demonstrated that the weekly total rainfall in the preceding 3 weeks best explained the increase in the OI and ODI of households and public areas, respectively, compared with the weekly total rainfall in the other lag week periods. This result may be helpful for encouraging the task force or local health authority to mobilize the local community to clean up the environment and empty water containers to reduce breeding sites. The present result is important in establishing an early warning system in each region of Indonesia.

### Implications and recommendations for vector surveillance policy

Dengue vector surveillance programs using the house index, container index, and Breteau index [6,7] have long been implemented in Indonesia. Local residents undertake such surveys on a regular basis under the coordination of local health authorities. However, the larval survey has not been well implemented. The chief difficulties in its implementation are the laborious procedure and that it largely depends on the surveyors’ discretion in larval surveying [9]. Low-level community participation, lack of incentives for local residents, and inconsistent data collection are major causes of failure in providing timely and accurate entomological data to local health authorities. The limitation is common throughout Indonesia. Periodic dengue outbreaks at our study site demonstrate that the dengue vector surveillance requires synchronized community mobilization for breeding source removal or other actions to control vector mosquitoes, which is helpful in improving the dengue epidemic [45–49].

Taken together, determining whether household ovitrap indices are significantly associated with monthly dengue cases and weekly rainfall in the preceding 3 weeks is crucial for expanding the use of ovitrap-based monitoring tools nationwide in Indonesia. This technique may be effective in monitoring the population dynamics of *Aedes* mosquitoes and predicting possible dengue outbreaks, and hence, it can be a valuable early warning indicator to initiate environmental clean-up before a dengue outbreak. In the present study, the receptivity of participating residents from both housing types to the study was high. However, challenges remained with regard to accessing to their properties, as some house owners were unavailable during visits, leading to visit rescheduling. If the survey covers a wide area and involves a high number of participating houses, this can be a potential problem. Given the positive correlations of OI and ODI between indoor and outdoor locations, we suggest that outdoor ovitraps may be sufficient for monitoring *A*. *aegypti* abundance in the urban community, as some residents are reluctant to allow inspectors access to their house. Data based on ovitrap indices of household evaluations are the most reliable but the least available if community participation is low. Alternatively, ovitrap placement in public areas that involves less human effort may be a solution to the low-level participation of local residents. Local health offices or community leaders may hire full-time surveyors to conduct routine inspections of ovitraps at outdoor or public areas. This method is convenient and easy for surveyors if they are tasked with a certain number of ovitraps at a designated area, unlike the existing larval surveillance methodology, where much effort is invested in searching for potential larval breeding sites and outcomes depend on surveyors’ discretion.

In this study, no significant difference in PHI and OI was observed between high-density housing and terraced housing. The socioeconomic status of local residents subtly influenced mosquito population dynamics at study sites, and the customary habit of storing water for daily use and bathing provides potential indoor breeding sites for *Aedes* mosquitoes. Our findings suggest that prevention measures, such as clearing natural and artificial water containers in public places and frequently checking water containers, including buckets and traditional bathtubs, should begin within 3–4 weeks after rains to reduce mosquito populations. To determine whether our results can be generalized to other suburban cities in Indonesia, further studies on ovitrap surveillance at other regions are required to validate the results. This is because the vectorial capacity of mosquitoes in a given area may vary locally, and thus, vector abundance might not necessarily reflect dengue transmission risk.

## Supporting information

S1 Fig Regression analysis of OI and ODI of outdoor and indoor household locations. (PDF)

S1 Table Summary of mean PHI, OI, and ODI values according to ovitrap placement at household/public and indoor/outdoor sites and housing type during wet, dry, and all seasons. (PDF)

S2 Table Household income (Table A) and education level (Table B) of respondents in terraced housing and high-density housing.
(PDF)
