# Supplementary figures and images for: Ovitrap surveillance of dengue vector mosquitoes in Bandung City, West Java Province, Indonesia
Source: PLoS Negl Trop Dis. 2021 Oct 28;15(10):e0009896. doi: 10.1371/journal.pntd.0009896 (PMC8577782; doi:10.1371/journal.pntd.0009896)

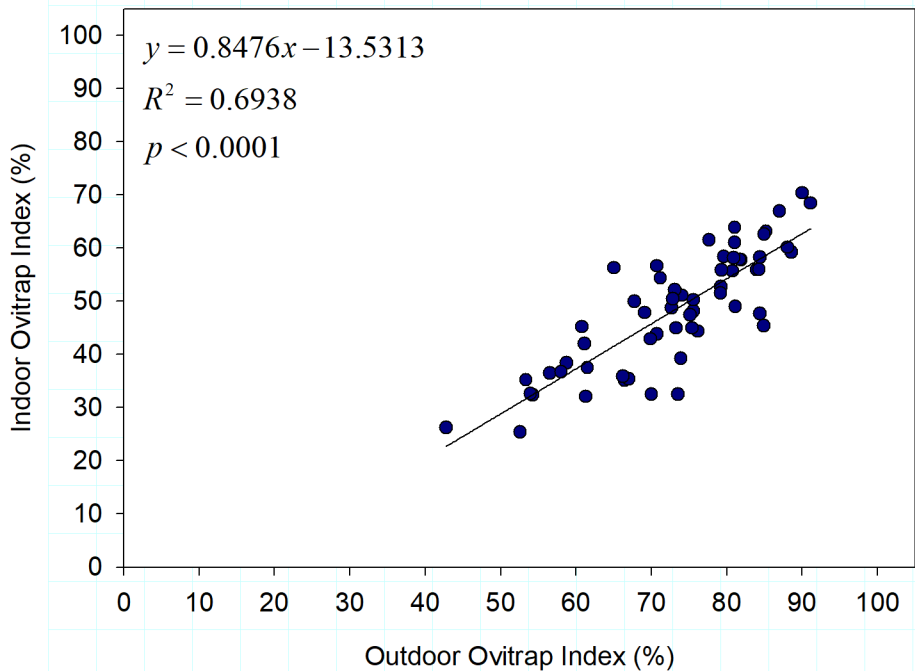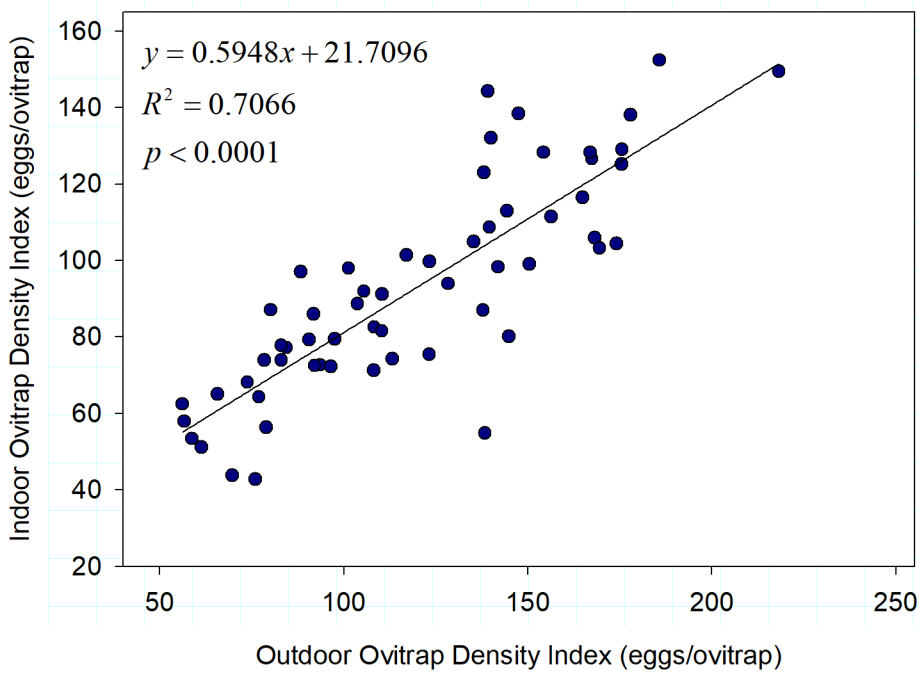

Supplement: S1 Fig — (PDF) [file pntd.0009896.s001.pdf]
