# Supplementary material for: Ovitrap surveillance of dengue vector mosquitoes in Bandung City, West Java Province, Indonesia
Source: PLoS Negl Trop Dis. 2021 Oct 28;15(10):e0009896. doi: 10.1371/journal.pntd.0009896 (PMC8577782; doi:10.1371/journal.pntd.0009896)
Supplement: S1 Table — (PDF) [file pntd.0009896.s002.pdf]

Table in S1 Table. Summary of mean PHI, OI, and ODI according to ovitrap placement at household/public and indoor/outdoor sites and housing type during wet, dry, and all seasons

Season	Mean PHI±SD (%)				Mean OI±SD (%)						Mean ODI±SD					
	Ovitrap placement (1)		Housing type		Ovitrap placement (1)		Ovitrap placement (2)		Housing type		Ovitrap placement (1)		Ovitrap placement (2)		Housing type	
	Household	Public	Terraced	High density	Household	Public	Indoor	Outdoor	Terraced	High density	Household	Public	Indoor	Outdoor	Terraced	High density
Dry	87.38±7.49	83.58±18.53	85.64±9.04	88.36±9.67	50.82±8.52	55.9±18.76	39.39±7.47	66.32±10.17	52.65±11.60	53.52±9.35	82.29±18.12	59.3±28.64	72.14±16.94	89.71±25.07	86.09±25.31	79.75±22.32
Wet	95.56±3.38	97.86±4.61	95.68±3.74	96.06±4.46	66.39±7.14	78.82±10.59	56.12±7.06	78.57±8.07	70.07±8.97	67.29±6.63	126.4±22.00	121.06±41.52	109.98±22.49	144.25±30.17	135.98±29.65	123.46±25.03
All season	91.85±6.96	91.32±14.73	91.09±8.34	92.54±8.22	59.27±11.00	68.33±18.72	48.46±11.06	72.96±10.91	62.09±13.41	60.99±10.52	106.22±29.95	92.80±47.45	92.66±27.58	119.29±38.98	113.15±37.22	103.46±32.26
