# Supplementary material for: Ovitrap surveillance of dengue vector mosquitoes in Bandung City, West Java Province, Indonesia
Source: PLoS Negl Trop Dis. 2021 Oct 28;15(10):e0009896. doi: 10.1371/journal.pntd.0009896 (PMC8577782; doi:10.1371/journal.pntd.0009896)
Supplement: S2 Table — Household income (Table A) and education level (Table B) of respondents in terraced housing and high-density housing. (PDF) [file pntd.0009896.s003.pdf]

S2A Table. Household income level, comparing respondents in Terraced housing and high density housing.

Income per month (IDR)	Housing type	Frequency	Statistical output
Below 3 million	Terraced	151	χ^2 = 32.571; df = 2; P < 0.0001
	High density	192	
Between 3 to 10 million	Terraced	101	
	High density	46	
Above 10 million	Terraced	8	
	High density	0	

S2B Table. Level of education, comparing respondents in Terraced housing and high density housing.

Level of education	Housing type	Frequency	Statistical output
Not finishing primary school	Terraced	3	χ^2 = 137.195; df = 4; P < 0.0001
	High density	8	
Primary school	Terraced	3	
	High density	56	
Junior high school	Terraced	20	
	High density	48	
Senior high school	Terraced	101	
	High density	103	
Higher education	Terraced	136	
	High density	25	
